# Chronic urticaria treatment patterns and changes in quality of life: AWARE study 2-year results

**DOI:** 10.1016/j.waojou.2020.100460

**Published:** 2020-09-12

**Authors:** Marcus Maurer, Ana Giménez-Arnau, Luis Felipe Ensina, Chia-Yu Chu, Xavier Jaumont, Paolo Tassinari

**Affiliations:** a Dermatological Allergology, Allergie-Centrum-Charité, Department of Dermatology and Allergy, Charité –Universitätsmedizin Berlin, Germany; b Dermatology Department, Hospital del Mar. IMIM, Universitat Autònoma, Barcelona, Spain; c CPAlpha Clinical Research Center, São Paulo, Brazil; d Hospital Sírio-Libanês, São Paulo, Brazil; e Department of Dermatology, National Taiwan University Hospital and National Taiwan University College of Medicine, Taipei, Taiwan; f Novartis Pharma AG, Basel, Switzerland

**Keywords:** Angioedema, Dermatology, Quality-of-life, Urticaria, AMAC, Asia-Pacific and the Middle East, AWARE, A Worldwide Antihistamine-Refractory Chronic Urticaria patient Evaluation, CIndU, chronic inducible urticaria, CSU, chronic spontaneous urticaria, CU, chronic urticaria, DLQI, dermatology life quality index, LaCAN, Central and Latin America, PRO, patient-reported outcome, QoL, quality of life, SD, standard deviation, UAS7, 7-day urticaria activity score

## Abstract

**Background:**

A Worldwide Antihistamine-Refractory Chronic Urticaria (CU) patient Evaluation (AWARE) is a non-interventional, multicenter study including patients from Europe, Central and Latin America, Asia-Pacific, and the Middle East. AWARE describes real-world evidence for CU, including clinical characteristics, treatment patterns and the impact on quality of life.

**Methods:**

Over the 2-year study, therapy changes, angioedema occurrence, and patient-reported outcomes (PROs) were recorded over 9 visits, including dermatology life quality index (DLQI) and 7-day urticaria activity score (UAS7). Data were stratified into subgroups: chronic spontaneous urticaria (CSU), chronic inducible urticaria (CIndU), or CSU + CIndU.

**Results:**

Out of 4838 patients analyzed, 9.9% were receiving no treatment for their CU symptoms at baseline, and 20.4% were receiving first-line non-sedating H_1_-antihistamine at approved doses. The predominant baseline therapy was up-dosed non-sedating H_1_-antihistamines (25.5%). By Visit 2, omalizumab was the overall most commonly used therapy (29.6%), increasing to 30.1% by the end of the study. Baseline DLQI scores for patients with CSU, CIndU and CSU + CIndU were 8.3, 7.6 and 9.1, respectively; scores decreased over the study for CSU and CSU + CIndU patients, but fluctuated for CIndU patients. Baseline angioedema occurrence was higher in CSU and CSU + CIndU patients, reported in 45.4% and 45.5% of patients, respectively, compared to 17.0% in CIndU patients. By the final visit, angioedema had decreased to 11.9% and 11.2% for CSU and CSU + CIndU, respectively, and 9.6% for CIndU.

**Conclusion:**

CU patients are undertreated at baseline; after entering the AWARE study, more patients received appropriate treatment. However, over two thirds are not escalated to third-line treatments.

## Introduction

Chronic urticaria (CU) is a heterogeneous group of skin diseases characterized by the rapid and recurrent appearance of itchy wheals, angioedema, or both, for longer than 6 weeks.^1^ CU has two subgroups: chronic spontaneous urticaria (CSU) and chronic inducible urticaria (CIndU). CSU is characterized by the spontaneous occurrence of wheals and/or angioedema, is more common than CIndU, and accounts for approximately two-thirds of CU cases.^2^ In CIndU, specific triggers such as cold temperatures induce wheals and angioedema (cold urticaria).^3^ Around 20% of patients with CU have a combination of CSU and CIndU (CSU + CIndU).^4^ With a point prevalence of 0.5–1% and a peak incidence in individuals between 20 and 40 years of age,^2^ CU is a common disorder that has a profound impact on patients’ quality of life (QoL).^5^, ^6^, ^7^

Historically, physicians have used H_1_-antihistamines as the standard of care in CSU.^1^^,^^8^^,^^9^ According to the current EAACI/GA^2^LEN/EDF/WAO guidelines for CU, the recommended first- and second-line therapies are standard-dosed and up-dosed (up to 4 times the approved dose) second-generation, non-sedating H_1_-antihistamine, respectively.^1^ However, many patients continue to experience CU signs and symptoms despite taking these treatments,^10^ with as many as 60% of patients with CSU not achieving symptom control at approved doses.^11^ The third-line treatment option is omalizumab, the only other licensed treatment for CSU. Several studies have demonstrated the efficacy and safety of omalizumab in CU;^1^^,^^12^, ^13^, ^14^, ^15^ additionally, omalizumab has over 15 years of clinical experience, and over 1.3 million patient-years of exposure (PSUR: Novartis Data on File as of December 31, 2019).

Our understanding of the causes and pathogenesis of urticaria is increasing;^16^, ^17^, ^18^ however, a need exists for global studies to gain greater understanding of the burden of disease, different treatment options used for CU, the lack of adherence to recommended guidelines,^19^ and the high rates of patients who are undertreated.^10^ To date, no worldwide study has been conducted to evaluate treatment decisions and the impact these decisions have on QoL in patients with CU. Previous data on the AWARE study in Europe, Central and Latin America (LaCAN)/Europe, and Asia-Pacific and the Middle East (AMAC) have been published.^20^, ^21^, ^22^ Our current study aims to investigate these questions from 3 regions across the world – Europe, LaCAN, and AMAC. We analyzed extensive pooled data from centers across the 3 AMAC regions to understand the impact different treatment options have on symptom control and QoL in H_1_-antihistamine-refractory patients with CSU, CIndU and CSU + CIndU, over a two-year period.

## Methods

### Study design

A Worldwide Antihistamine-Refractory CU patient Evaluation (AWARE) is a pooled non-interventional study that comprises 3 worldwide multicenters (Europe, LaCAN, and AMAC), which includes first-line H_1_-antihistamine-refractory patients with CSU, CIndU, and CSU + CIndU from real-life clinical settings. Detailed study designs for each region have previously been reported.^10^ Over the two-year study period (across 9 visits each spaced at around 3 monthly intervals), data were collected to analyze the effects of different treatments on symptom control and improvement in QoL. Baseline therapy and therapy changes were also recorded. To analyze changes over time, patient-reported outcomes (PROs) including dermatology life quality index (DLQI [scale 0–30, with higher worse]) and 7-day urticaria activity score (UAS7 [scale 0–42, with higher worse]) were recorded, as well as angioedema occurrence. Data were stratified according to diagnostic group: CSU, CIndU, and CSU + CIndU.

Patients had a baseline Visit (Visit 1) and 8 follow-up visits in roughly quarterly intervals. Average visits relate to the following timeframes: Visit 1, 0 months (Day 1, baseline); Visit 2, 3 months; Visit 3, 6 months; Visit 4, 9 months; Visit 5, 12 months; Visit 6, 15 months; Visit 7, 18 months; Visit 8, 21 months; and Visit 9, 24 months.

The institutional review board of each participating center approved the study protocol. The trial was conducted in accordance with the Declaration of Helsinki and Good Clinical Practice and in compliance with all federal, local, and regional requirements. The manufacturer of omalizumab sponsored AWARE.

### Inclusion and exclusion criteria

The inclusion criteria were as followed: patients aged ≥18 years, who provided written informed consent, a medically-confirmed diagnosis of CU (defined as recurrent episodes of wheals [hives], angioedema, or both) for at least 2 months, presence of signs and/or symptoms of CU, and patients must have tried at least one course of H_1_-antihistamines (at any approved dose) for 2 weeks and been refractory to this treatment. Patients who were involved in or planned to participate in another interventional clinical study for CU were excluded.

### Patients

Data from 5223 patients enrolled from urticaria centers and office-based dermatological practices across the 3 study regions were pooled, including 3741 from Europe, 989 from AMAC, and 493 from LaCAN. In total, 385 patients discontinued the study due to not meeting the study criteria. Of the remaining 4838 patients included in this analysis, those with CSU made up the majority of the population (3,387, 70%), followed by patients with CSU + CIndU (1,203, 24.9%) and CIndU (248, 5.1%, Table 1).

**Table 1:** Baseline demographics and disease characteristics.

	CSU N = 3387	CIndU N = 248	CSU + CIndU N = 1203	Overall N = 4838
Age, years (SD)	45.5	41.3	42.5	44.6 (15.2)
Gender, Female (%)	71.6%,	69.8%	73.0%	71.8
n (%)	3387 (70.0)	248 (5.1)	1203 (24.9)	4838 (100.0)
Angioedema^a^, n (%)	1527 (45.4)	42 (17.0)	547 (45.5)	2116 (44.0)
Time since diagnosis, years (SD)	4.7 (7.1)	6.0 (7.9)	5.7 (7.3)	5.0 (7.2)
DLQI (SD)	8.3 (6.9)	7.6 (6.7)	9.1 (6.9)	8.5 (6.9)
UAS7 (SD)	17.6 (12.3)	N/A	N/A	N/A

a Number of patients with angioedema within the previous 6 months of baseline is recorded. DLQI, Dermatology Life Quality Index; n, number of patients; N/A, not available; SD, standard deviation; UAS7, urticaria activity score

This study was designed and implemented in accordance with the ethical principles in the Declaration of Helsinki.

### Patient-reported outcomes

The once-daily Urticaria Activity Score (UAS7) and Dermatology Life Quality Index (DLQI) were used to assess disease activity and impact on patients’ QoL, respectively. UAS7 assesses disease activity with 2 questions about wheals and itching over 7 consecutive days, which ranges from 0 (no disease activity/complete response to treatment) to 42 (highest possible disease activity).^23^ DLQI is a validated, dermatology-specific 10-item questionnaire covering 6 areas: symptoms and feelings; daily activities; leisure; work and school; personal relationships; and effects of treatment on daily life. The DLQI score ranges from 0 (highest QoL) to 30 (lowest QoL).^24^

### Statistical analysis

The results herein are a pooled analysis of 3 non-interventional studies from Europe, LACan, and AMAC, and they are reported as observed. Quantitative data are reported as means, medians, standard deviation (SD), minimum (0%) and maximum (100%). Absolute and relative data are reported as means, based on the number of patients fulfilling the respective condition. Results are also reported as percentage of patients, minimum (0%) and maximum (100%).

## Results

### At baseline, 1 in 10 patients were receiving no treatment, and only 1 in 2 patients were receiving first- or second-line CU treatment

Out of 4838 total patients, 480 (9.9%) patients were receiving no treatment for their CU symptoms at baseline, and only 986 (20.4%) were receiving the recommended first-line non-sedating H_1_-antihistamine at approved doses. The most commonly used therapy at baseline was second-line up-dosed non-sedating H_1_-antihistamines, which was used by 1236 (25.5%) patients (Table 2). Other treatments that patients used at baseline consisted of omalizumab, montelukast, and ciclosporine (Table 2).

**Table 2:** Urticaria therapies overall and by diagnostic subgroup at baseline and at study end.

Diagnostic subgroup	CSU		CIndU		CSU + CIndU		Overall	
	Baseline (V1)	Study end (V9)	Baseline (V1)	Study end (V9)	Baseline (V1)	Study end (V9)	Baseline (V1)	Study end (V9)
NS H_1_-antihistamine approved, n (%)	716 (21.1)	255 (14.5)	48 (19.4)	13 (10.3)	222 (18.5)	107 (15.8)	986 (20.4)	375 (14.7)
Up-dosed NS H_1_-antihistamine, n (%)	866 (25.6)	236 (13.4)	81 (32.7)	28 (22.2)	289 (24.0)	88 (13.0)	1236 (25.5)	352 (13.8)
On demand NS H_1_-antihistamine, n (%)	180 (5.3)	189 (10.8)	18 (7.3)	17 (13.5)	65 (5.4)	59 (8.7)	263 (5.4)	265 (10.4)
S H_1_-antihistamine, n (%)	115 (3.4)	49 (2.8)	12 (4.8)	2 (1.6)	36 (3.0)	17 (2.5)	163 (3.4)	68 (2.7)
Combination NS H_1_-antihistamine & S H_1_-antihistamine, n (%)	148 (4.4)	52 (3.0)	10 (4.0)	1 (0.8)	78 (6.5)	29 (4.3)	236 (4.9)	82 (3.2)
Omalizumab^a^, n (%)	720 (21.3)	523 (29.8)	32 (12.9)	34 (27.0)	273 (22.7)	212 (31.4)	1025 (21.2)	769 (30.1)
Montelukast^b^, n (%)	180 (5.3)	37 (2.1)	10 (4.0)	7 (5.6)	85 (7.1)	26 (3.8)	275 (5.7)	70 (2.7)
Ciclosporine^c^, n (%)	34 (1.0)	10 (0.6)	1 (0.4)	1 (0.8)	13 (1.1)	6 (0.9)	48 (1.0)	17 (0.7)
Other, n (%)	87 (2.6)	48 (2.7)	9 (3.6)	2 (1.6)	30 (2.5)	19 (2.8)	126 (2.6)	69 (2.7)
No treatment, n (%)	341 (10.1)	358 (20.4)	27 (10.9)	21 (16.7)	112 (9.3)	113 (16.7)	480 (9.9)	492 (19.2)
**Total, n (%)**	**3387 (100.0)**	**1757 (100.0)**	**248 (100.0)**	**126 (100.0)**	**1203 (100.0)**	**676 (100.0)**	**4838 (100.0)**	**2559 (100.0)**

a Treatment with omalizumab (without ciclosporine).

b Treatment with montelukast (without ciclosporine or omalizumab).

c Treatment with ciclosporine (without montelukast or omalizumab) n = number of patients; CIndU, chronic inducible urticaria; CSU, chronic spontaneous urticaria; NS, non-sedating; S, sedating

### The use of standard and up-dosed H1-antihistamines decreased over the two-year study period

**Fig. 1:**
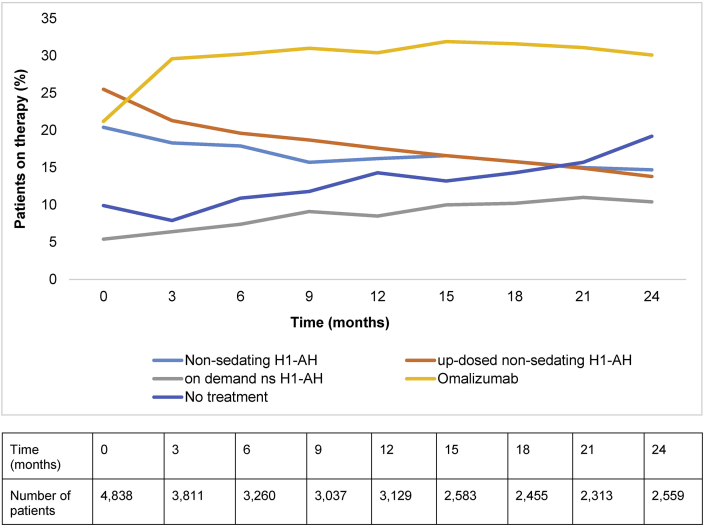
**Therapy changes over time.** ∗Therapy at baseline (Visit 1, Day 1) is defined as every documented therapy with a start date prior or equal to baseline and ongoing at baseline. Visit 9 is approximately 24 months after baseline and defines the end of study. The table below the graph shows the total number of patients who remained in the study throughout the 2-year period. AH, antihistamine.

The use of up-dosed non-sedating H_1_-antihistamine consistently decreased throughout the two-year study period, and by the end of the study (Visit 9) was used by 352 (13.8%) of the remaining 2559 patients (Fig. 1). A similar trend was observed for patients treated with the standard dose of non-sedating H_1_-antihistamine therapy, which decreased to 375 (14.7%) patients by the end of the study (Fig. 1). Overall, the proportion of patients either on no treatment, first-, second or third-line treatments did not differ between baseline (77%) and the end of the study (77.8%; Table 2).

### Omalizumab use increased from baseline to become the most commonly used treatment throughout the study

Overall, 720/4838 (21.2%) patients were using omalizumab at baseline, which increased to 769/2559 (30.1%) patients by the end of the study. There was an increase in its use by Visit 2 to 1128/3811 (29.6%), and it remained the most commonly used treatment throughout the study (Fig. 1).

### Nearly half of all patients had angioedema at baseline, which decreased to 1 in 10 by the end of the study

**Fig. 2:**
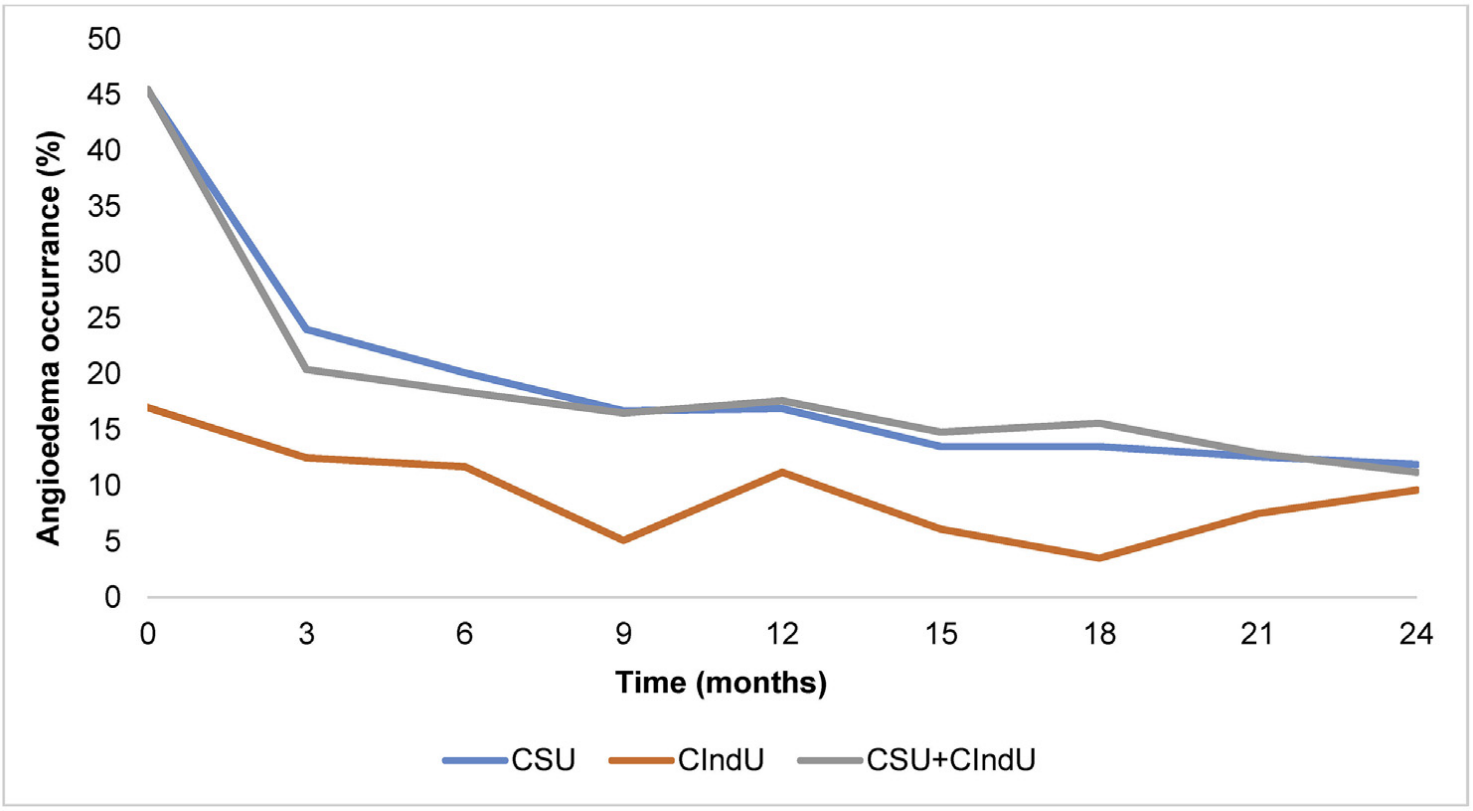
**Current angioedema or angioedema during the last 6 months or 12 weeks throughout the study.** Percentages of the total population per diagnostic group are shown for patients with angioedema. CSU, chronic spontaneous urticaria; CIndU, chronic inducible urticaria

At baseline, 2116/4812 (44.0%) patients reported angioedema (Table 1); those with CSU (1527/3,363, 45.4%) and CSU + CIndU (547/1,202, 45.5%) had the highest occurrences of angioedema. By the end of the study, angioedema had decreased to 11.9% (179/1502) and 11.2% (55/490) for CSU and CSU + CIndU, respectively, with a consistent decline throughout the entire study period (Fig. 2). Comparatively, only 17.0% (42/247) patients with CIndU reported having angioedema at baseline, which decreased to 9.6% (11/115) by the end of the study. Although angioedema occurrence remained low in patients with CIndU compared to those with CSU and CSU + CIndU, the percentage of patients with angioedema fluctuated throughout the two-year study period (Fig. 2).

### DLQI scores of 0/1 steadily increased throughout the study period in all diagnostic groups

**Fig. 3:**
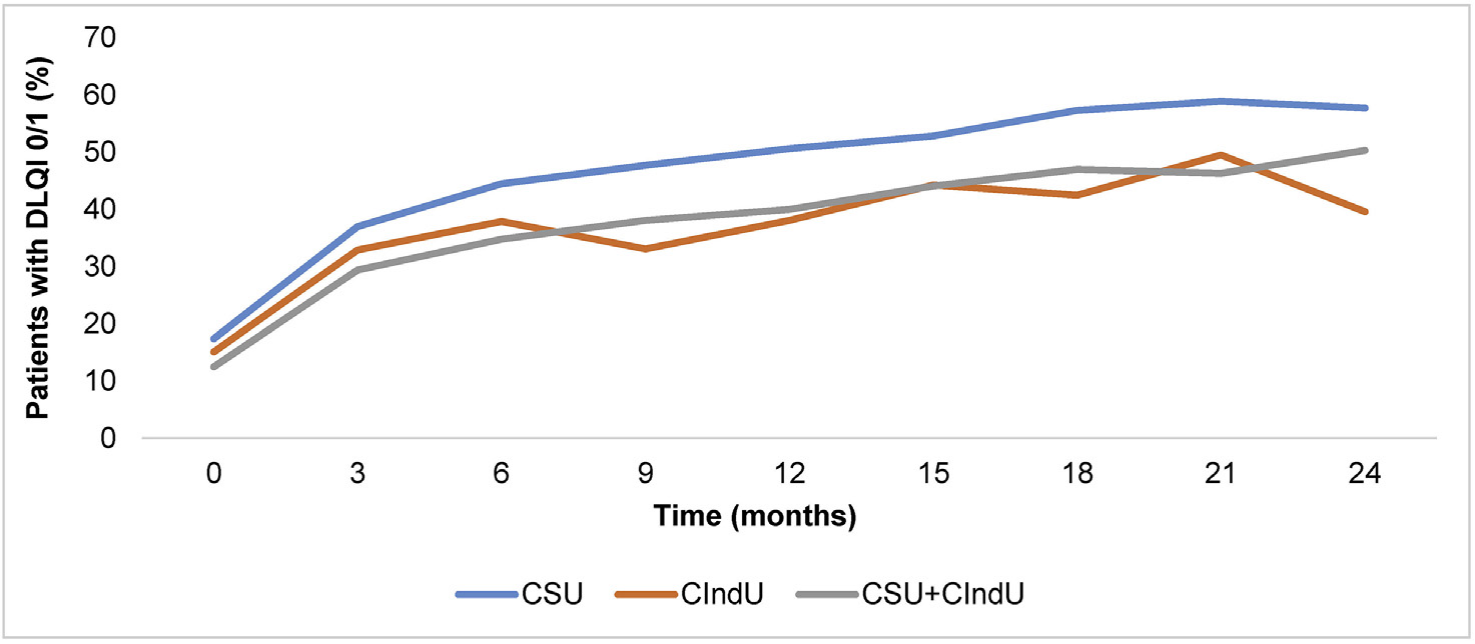
**Mean percentage of patients achieving Dermatology Life Quality Index 0/1 per diagnostic group throughout the study.** Changes over time were reported in the percentage of patients achieving a DLQI score of 0/1 (scale 0–30, with higher worse). CSU, chronic spontaneous urticaria; CIndU, chronic inducible urticaria; DLQI, Dermatology Life Quality Index

The overall baseline DLQI score was 8.5 (Table 1). At baseline, the percentage of patients with a DLQI score of 0/1 was 17.4% (569/3275), 15.1% (37/245), and 12.5% (147/1172) for CSU, CIndU, and CSU + CIndU, respectively. This increased consistently over the 2-year study period for patients with CSU and CSU + CIndU, but fluctuated for patients with CIndU (Fig. 3). Patients with CSU achieved the highest proportion of DLQI scores of 0/1 throughout the entire 2-year study, increasing consistently until patients achieved a maximum of 58.9% (854/1451) at Visit 8 (21 months). The percentage of patients with CSU + CIndU achieving a DLQI score of 0/1 also increased consistently to a maximum of 50.3% (232/461) by the end of the study. For patients with CIndU, 49.5% (48/97) achieved a DLQI score of 0/1 at Visit 8; however, the increasing trend fluctuated unlike in patients with CSU and CSU + CIndU (Fig. 3).

### UAS7 scores improved in patients with CSU throughout the study

**Fig. 4:**
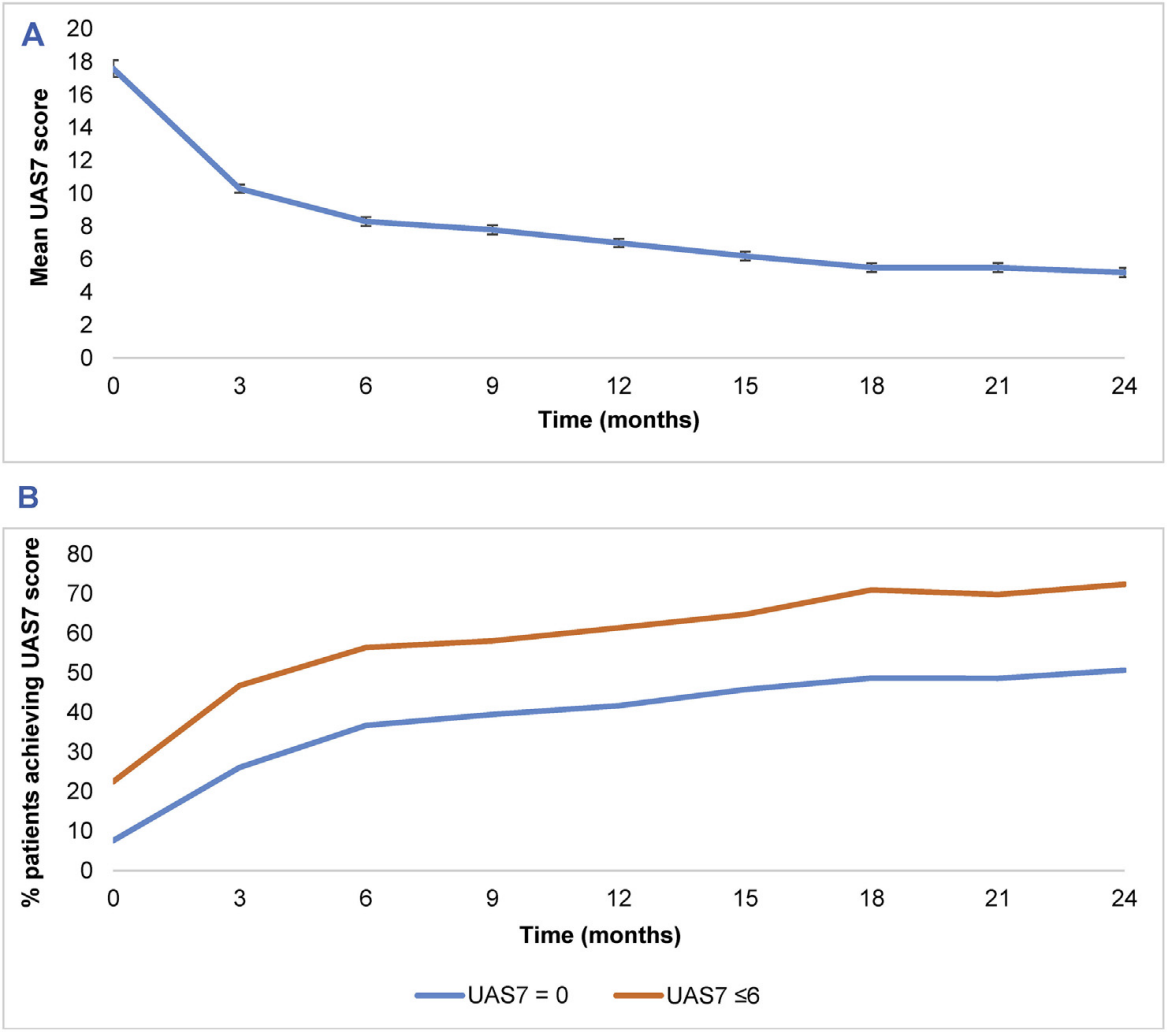
**7-Day urticaria activity scores (UAS7) in patients with chronic spontaneous urticaria throughout the study. A/Mean UAS7 scores and B/Percentage of patients achieving a UAS7 score of 0 or** ≤ **6.** Changes over time were measured using the 7-day urticaria activity score (scale 0–42, with higher worse) at each visit. A. shows the mean overall UAS7 score of all patients and B. shows the percentage of patients achieving UAS7 scores of 0 and ≤ 6. Error bars in graph A represent the standard error. UAS7, 7-day urticaria activity score

The mean baseline UAS7 score in patients with CSU was 17.6 (Table 1). This decreased consistently throughout the two-year study with patients achieving the lowest mean UAS7 score of 5.2 by the end of the study (Fig. 4 A and B). Additionally, UAS7 scores continually improved throughout the study in patients with CSU from baseline to the end of the study, as demonstrated by the percentage of patients achieving a score of 0 (from 7.6% to 50.7%), and those achieving a score of ≤6 (from 22.5% to 72.4%).

## Discussion

The non-interventional AWARE study includes 4838 patients from Europe, LaCAN, and AMAC, and provides valuable data on treatment patterns in patients with CU across the world. Variations in treatment options for CU were observed throughout this two-year study, alongside improvements in PROs across all diagnostic subgroups. Although no direct correlations can be made with non-interventional studies, the sustained improvement in PROs confirms previous reports that effective CU treatments can improve QoL and reduce disease severity and the occurrence of angioedema in H_1_-antihistamine-refractory patients.^14^^,^^25^^,^^26^

At baseline, as many as 1 in 10 patients reported receiving no CU treatment, and only 1 in 5 patients were receiving the recommended first-line treatment of standard-dose H_1_-antihistamines. Follow-up data suggests that, over 2 years of treatment, less than 1 in 3 patients who may have benefitted from being moved to the more effective third-line treatment were actually switched to that treatment; this of course, could also have been due to funding issues or patient preferences.

All analyzed patients had medically confirmed CU; it is possible that some of the patients who were not receiving any CU treatment had experienced a long period between disease onset and diagnosis, and therefore they did not receive appropriate treatment in a timely manner. Availability and reimbursement of omalizumab is likely to differ between participating centers, and indeed, within continents. The current study was intended to provide an overall picture of treatment patterns and their outcomes by pooling the 3 regions, thereby maximising the population size. Results presented herein can help inform future non-pooled studies that compare different regions on socioeconomic issues, which influence treatment choices. We note that omalizumab for the treatment of CIndU is off-label, and evidence for its efficacy in CU mainly comes from its use in patients with CSU.^15^ We suggest more detailed studies would help determine clinicians’ treatment choices.

The most frequently used therapy at baseline was up-dosed H_1_-antihistamines. By Visit 2, many patients had been switched to the next treatment step, as per the guidelines. The continuous decline in the use of H_1_-antihistamine and up-dosed H_1_-antihistamine throughout the study period suggests that first- and second-line treatments were ineffective in controlling CU symptoms.

The increase in patients who reported receiving no treatment from baseline to the end of the study could be due to many reasons such as spontaneous remission, or the fact that physicians were unaware of CU treatment guidelines and options. This supports results from previous studies that have highlighted the challenges currently faced in CU disease management.^27^ Because CU is a self-limiting disease,^28^ the decrease in treated patients could be due to natural improvements in disease activity, or because patients are part of a protocol with regular follow-up visits. This inherent limitation of CU causes challenges in data interpretation. Our study highlights the high number of patients who, by the end of a two-year follow-up period, have not received the appropriate, effective treatment to tackle their CU.

The overall number of patients reporting angioedema declined for all CU subgroups indicating that the treatment strategies over the previous 2 years were effective in controlling angioedema, especially in patients with CSU and CSU + CIndU. The occurrence of angioedema is higher in patients with CSU or CSU + CIndU, with a baseline incidence of over 45% patients in both subgroups compared to 17% in patients with CIndU. Patients with CIndU reported the lowest levels of angioedema, which may be reflective of the subtle differences in pathophysiology between the subgroups. Angioedema has been shown to detrimentally affect disease severity and patients’ QoL,^12^ and it is reported to correlate with the highest CSU activity (UAS7);^29^ this is reflected in the baseline DLQI scores of each diagnostic subgroup in this study, which show that patients with CSU and CSU + CIndU both reported the highest DLQI scores of 8.3 and 9.1, respectively, compared to 7.6 in patients with CIndU.

Overall, patients' DLQI scores in all subgroups decreased throughout the two-year study period with more patients with CSU achieving a DLQI score of 0/1 than in any other group. However, the percentage of patients achieving DLQI 0/1 in the CIndU group fluctuated and was similar to the fluctuations observed in the angioedema occurrence in patients with CIndU. This reflects the impact of angioedema on patients’ DLQI scores and highlights the importance of controlling angioedema occurrence during CU management.

Baseline UAS7 scores for patients with CSU (17.6) suggest these patients had moderate disease severity, over which they had achieved good control by Visit 6 (6.2) and maintained until the end of the study (5.2). Results from this study also suggest that patients with CSU are more likely to have steady improvements over time, unlike the fluctuating trends observed in patients with CIndU and CSU + CIndU, indicating that disease management is consistent in patients with CSU.

Prospective, non-interventional studies have inherent limitations: due to the range of centers from which patients were recruited, there is a lack of consistency in available treatments, eg, third-line omalizumab treatment is not generally available in the community, and therefore this will have influenced the final results. Additionally, the severity of CSU may be affected by many factors, including physical and psychological stress, and associated allergic disease, which we did not measure in the current study.

In summary, it appears that a high proportion of patients with CU are not receiving any treatment for their condition, and as few as 1 in 5 patients receive the recommended first-line treatment of standard-dose H_1_-antihistamines. Additionally, less than 1 in 3 patients who should be moved to a more effective third-line treatment are actually switched to that treatment, thereby indicating the possible need for further education of clinicians in the management of CU. Global multicenter studies are required to help highlight the discrepancies in CU management and inform clinicians of effective and reliable treatment strategies for all CU patients.

## Ethics statement

The authors declare that this manuscript complies with the ethics in publishing guidelines.

## Author contributions statement

The authors declare that they have made substantial contributions to the conception and design of the study, acquisition of data, or analysis and interpretation of data, of drafting the article or revising it critically for important intellectual content, and all authors have provided approval of the final version submitted.

## Consent for publication and availability of data and materials statement

Novartis consent to the publication of these data and are committed to data sharing, where appropriate.

## Funding statement

This investigation was sponsored by Novartis Pharma AG, Basel, Switzerland.

## Declaration of competing interest

**M. Maurer** has received grant/research support and/or honoraria for consulting or lectures from Aralez, Allakos, FAES, Genentech, Merckle Recordati, Moxie, Novartis, Roche, Sanofi, MSD, UCB, Uriach. **A. Giménez-Arnau** has served as medical advisor for Uriach Pharma, Genentech, Novartis, FAES, GSK, Sanofi, and received research grants supported by Uriach Pharma, Novartis, Grants from Instituto Carlos III- FEDER and has been involved in educational activities for Uriach Pharma, Novartis, Genentech, Menarini, LEO Pharma, GSK, MSD, Almirall and Sanofi. **LF Ensina** has received advisory board, speaker and investigator fees from Novartis, and speaker fees from Takeda. **C–Y Chu** is a clinical trial investigator for Novartis and has received travel support, consulting fees and payment for lectures from Novartis. **X. Jaumont** and **P. Tassinari** are employees of Novartis.
